# Zero Fluoroscopy Arrhythmias Catheter Ablation: A Trend Toward More Frequent Practice in a High-Volume Center

**DOI:** 10.3389/fcvm.2022.804424

**Published:** 2022-04-28

**Authors:** Federica Troisi, Pietro Guida, Federico Quadrini, Antonio Di Monaco, Nicola Vitulano, Rosa Caruso, Rocco Orfino, Giacomo Cecere, Matteo Anselmino, Massimo Grimaldi

**Affiliations:** ^1^Cardiology Department, Regional General Hospital “F. Miulli”, Bari, Italy; ^2^Department of Clinical and Experimental Medicine, University of Foggia, Foggia, Italy; ^3^Division of Cardiology, Department of Medical Sciences, “Città della Salute e della Scienza di Torino” Hospital, University of Turin, Turin, Italy

**Keywords:** arrhythmia, catheter ablation, efficacy, feasibility, fluoroscopy, safety, zero-fluoroscopy

## Abstract

**Background:**

Awareness of radiation exposure risks associated to interventional cardiology procedures is growing. The availability of new technologies in electrophysiology laboratories has reduced fluoroscopy usage during arrhythmias ablations. The aim of this study was to describe procedures with and without X-Rays and to assess feasibility, safety, and short-term efficacy of zero fluoroscopy intervention in a high-volume center oriented to keep exposure to ionizing radiation as low as reasonably achievable.

**Methods:**

Cardiac catheter ablations performed in our hospital since January 2017 to June 2021.

**Results:**

A total of 1,853 procedures were performed with 1,957 arrhythmias treated. Rate of fluoroless procedures was 15.4% (285 interventions) with an increasing trend from 8.5% in 2017 to 22.9% of first semester 2021. The most frequent arrhythmia treated was atrial fibrillation (646; 3.6% fluoroless) followed by atrioventricular nodal reentrant tachycardia (644; 16.9% fluoroless), atrial flutter (215; 8.8% fluoroless), ventricular tachycardia (178; 17.4% fluoroless), premature ventricular contraction (162; 48.1% fluoroless), and accessory pathways (112; 31.3% fluoroless). Although characteristics of patients and operative details were heterogeneous among treated arrhythmias, use of fluoroscopy did not influence procedure duration. Moreover, feasibility and efficacy were 100% in fluoroless ablations while the rate of major complications was very low and no different with or without fluoroscopy (0.45 vs. 0.35%).

**Conclusion:**

Limiting the use of X-Rays is necessary, especially when the available technologies allow a zero-use approach. A lower radiation exposure may be reached, reducing fluoroscopy usage whenever possible during cardiac ablation procedures with high safety, full feasibility, and efficacy.

## Introduction

In the last 15 years, the awareness of the harmfulness of radiation used in interventional cardiology and to guide percutaneous procedure has increased. In the electrophysiological field, the development of technologies has reduced progressively fluoroscopy guidance for catheter placement during arrhythmia ablations. This all began with the birth of electroanatomical mapping systems, that allow visualization of catheters used during ablation without radiation. These systems are based on three-dimensional reconstructions of cardiac chamber and on visualization of catheters that navigate inside. Progressively over the years, new technological advances in three-dimensional (3D) electroanatomical mapping (EAM) have been introduced both as diagnostic algorithms and as structural characteristics of the catheters (1, 2). It became possible in this way to reduce fluoroscopy time until zeroing, because new algorithms facilitate the recognition of the mechanisms of arrhythmias and the localization of their site of origin. On the other hand, structural improvements of the catheters increase safety of navigation and ablation without scope (3–5).

The American College of Cardiology recommends that all catheterization laboratories adopt the principle of “ALARA:” radiation doses to be used are “As Low as Reasonably Achievable” (6). This suggestion derived from knowledge of the biological effects of radiations both on medical staff and on patients; from the interaction with organisms, X-Rays create hydroxyl radicals that can damage the DNA of cells with pro-carcinogenic effects.

In our electrophysiology laboratory, we have progressively implemented measures to reduce the use of X-Rays during procedures. For 10 years we have been carrying out zero fluoroscopy ablation procedures, at the beginning mainly on supraventricular or ventricular ablations on the right heart, then progressively other, more complex, and procedures. Our hospital today is a high-volume procedure center, with considerable experience in zero X-Rays ablations.

According to recent data from a European multicenter registry, ~7% of procedures are conducted without any use of fluoroscopy while procedural settings (i.e., 3D-mapping system) and higher case volumes (7) are associated with a reduced usage of fluoroscopy. Data from high-volume electrophysiology laboratories that have progressively implemented measures to reduce the use of X-Rays during procedures are lacking. The aim of this study was: (1) to describe the contemporary use of fluoroscopy in an interventional electrophysiology laboratory; (2) to assess the proportion of procedures conducted without any use of fluoroscopy during catheter ablation of different arrhythmias; and (3) to evaluate feasibility, safety, and short-term efficacy of zero fluoroscopy intervention in real life.

## Methods

We conducted a retrospective analysis based on the Cardiac Interventional Registry implemented at our hospital (approval number 5690 by the Ethical Review Authority of the Azienda Universitaria Ospedaliera Consorziale - Policlinico, Bari), considering catheter ablations performed in our laboratory with and without fluoroscopy since January 2017 to June 2021. For each performed procedure, we classified the ablation according to arrhythmia/arrhythmias treated: atrioventricular nodal reentrant tachycardia (AVNRT), accessory pathways (AP), atrial fibrillation (AF), atrial flutter (ALF), premature ventricular contraction (PVC), and ventricular tachycardia (VT).

All procedures were performed with the CARTO3 mapping system (Biosense Webster, Irvine, California, USA) as the main imaging modality to cardiac chambers' navigation and catheter ablation. The CARTO3 system is based on electromagnetic technology: it uses low electromagnetic fields to identify the position of the catheter. Nine coils positioned under the patient bed generate three different electromagnetic fields, allowing the creation of a strong magnetic field less sensitive to distortion potentially created by proximity to fluoroscopy. Navistar sensor-based catheters contain three magnetic sensors in the distal part of the tip that allow the exact location within the magnetic field to be created.

The choice to perform intervention with or without X-Rays was made subjectively by the operator before starting the procedure. Only in AF was the decision not to use X-Rays made during the procedure, based on the possibility of passing with the catheters in the left atrium through the patent foramen ovale (PFO), thus avoiding the phase of the transeptal puncture.

### Fluoroscopy Procedures

From 2017, most procedures were performed with the integrated use of X-Rays and EAM, which reduces time of fluoroscopy compared to that of the ablative procedures used a few years ago. During fluoroscopy, ablation patients are in conscious sedation; we use a maximum of two femoral accesses for procedure. Ablation were carried out as previously described (8, 9).

### Fluoroless Procedures

No additional pre-procedure radiologic imaging was performed. The procedural workflow included the following steps: common right femoral vein puncture performed echo-guided to avoid casual puncture of artery or superficial femoralvein, insertion of diagnostic multi-electrode catheter sensor based (Decanav, Biosense Webster, Irvine, California, USA) or an ablation catheter, a 3.5 mm externally irrigated radiofrequencies with contact force sensor (Thermocool Smarttouch; Biosense Webster, Irvine, California, USA); compensation for respiratory movements; and advancement of the catheter both using tactile feedback and creating a calibration matrix of the inferior vena cava by sweeping the catheter tip whilst advancing it up to the initial appearance of atrial electrograms. In this way we identify the junction between the inferior vena cava, the right atrium, and the superior vein cava and mark them with a snapshot of the catheter. Then we create 3D geometric contours of the right atrial with fast anatomical mapping software with the aim of defining endocardial boundaries, tagging the area where a His bundle deflection was recorded, reproducing in detail the tricuspid annulus, and reconstructing the coronary sinus up to its distal portion. Finally, we advance the other diagnostic catheters, if needed, using the previously reconstructed venous and atrial geometry. The same technique is used for arterial access in case of a retrograde approach for left PVC, AP, or VT ablation.

### AVNRT Fluoroless Ablation

**Figure 1:**
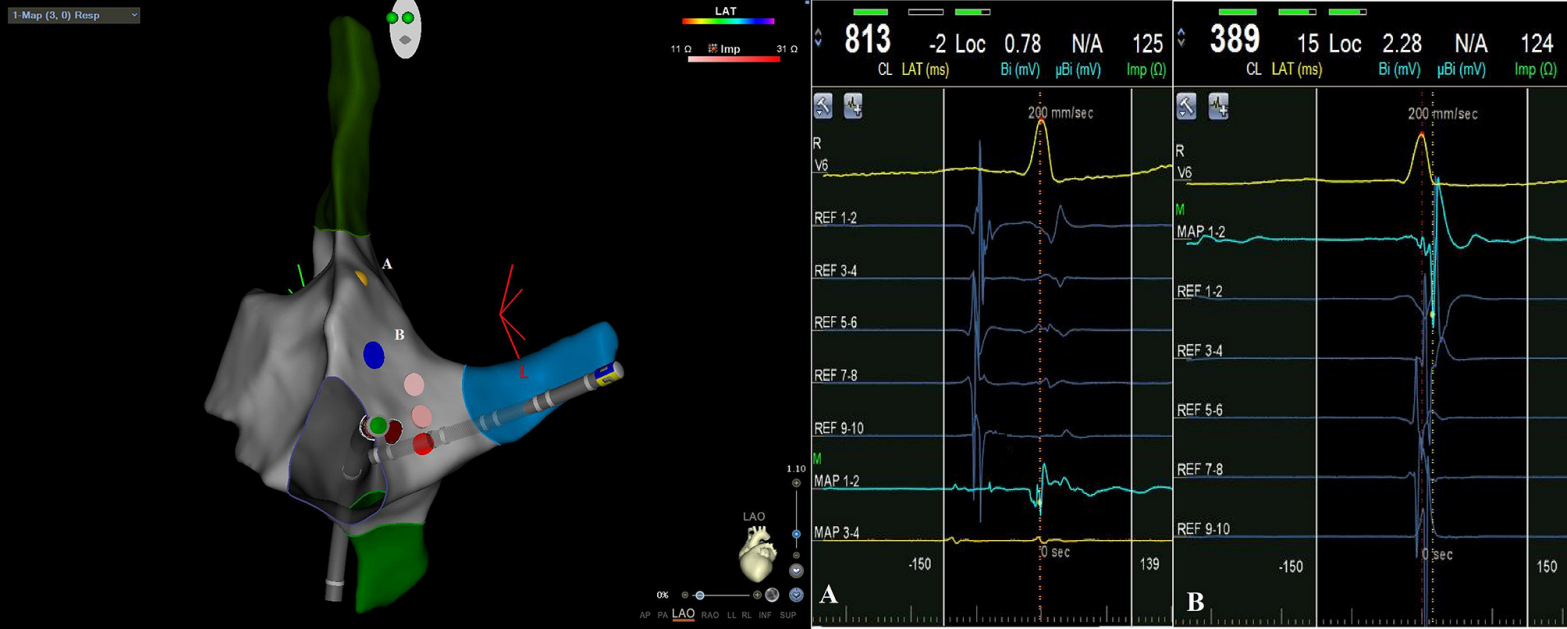
CARTO3 left anterior oblique (LAO) image of atrioventricular nodal reentrant tachycardia ablation: during radiofrequency applications catheter remains below tagged His potential [yellow point A on EAM, intracavitary signal **(A)**] and fast way potential [blue point B on EAM, intracavitary signal **(B)**].

When we arrive with catheters in the right atrium we identify and tag His potential; then we locate decapolar catheter in the coronary sinus. After inducing tachycardia by electrophysiological study, we carefully map slow pathway potential with ablation catheter, identifying and tagging the fast pathway potential. Then, always remaining below the tagged fast pathway potential, we start radiofrequency applications (Figure 1). Acute success was non-inducibility of tachycardia and evidence of a jump during programmed atrial stimulation with only one re-entry.

### AP Fluoroless Ablation

For the left-sided accessory pathway, we used a transaortic retrograde approach. We map the accessory pathway with ablation catheter, having as anatomical reference a decapolar catheter positioned in the coronary sinus. We look for Kent's potential or, in any case, for the point of greatest fusion between the atrial and ventricular potential in sinus rhythm for manifest AP, during continuous ventricular pacing for occult accessory pathways. Acute success was non-inducibility of tachycardia and for manifest AP elimination of antegrade accessory pathway conduction.

### AF Fluoroless Ablation

In our center we have used for several year a “near zero X-Rays” approach for transeptal puncture, which is the step that needs greater fluoroscopy during AF ablation procedure; this strategy was illustrated in a previous paper (10). In these procedures we did not use X-Rays at all because, during mapping of fossa ovalis it was found PFO, so we could go through to the left atrium without transeptal puncture. Once in the left atrium with the ablation catheter and the diagnostic catheter, we proceeded with the electroanatomical map guided by EAM and with the subsequent ablation procedure (pulmonary vein isolation and other ablative targets). Acute success was pulmonary veins isolation validation and non-inducibility of AF.

### AFL Fluoroless Ablation

When catheters arrive in the right atrium, first of all we verify the location of the circuit with entrainment on vena cava tricuspid isthmus. If it is a typical AFL, we are already at the right place and we go to ablation. If not, we start mapping atrial arrhythmia using both electrophysiological potentials and activation map provided by EAM. Validation of bidirectional block with pacing stimulation and non-inducibility of tachycardia during electrophysiological tests was considered as acute success.

### PVC and VT Fluoroless Ablation

Depending on the origin of PVC/VT diagnosed by the electrocardiogram, we use a venous or arterial approach. Once arrived with the catheters in the heart chamber of interest, we start mapping PVC/VT with activation and simultaneously substrate map processed with EAM. Complete elimination of PVC or non-inducible VT during ventricular stimulation was acute success of cardiac ablation.

### Statistical Analyses

Data are summarized as the mean ± standard deviation for continuous variables or frequency and percentage for categorical variables. Characteristics of patients treated with and without fluoroscopy were compared by using Student's *t*-test or chi-squared test (respectively for continuous and categorical data) while Fisher's exact test was applied for the rate of major complications. *P*-value < 0.05 was considered to be statistically significant. All analyses were performed using STATA version 16 (StataCorp, College Station, Texas).

## Results

**Figure 2:**
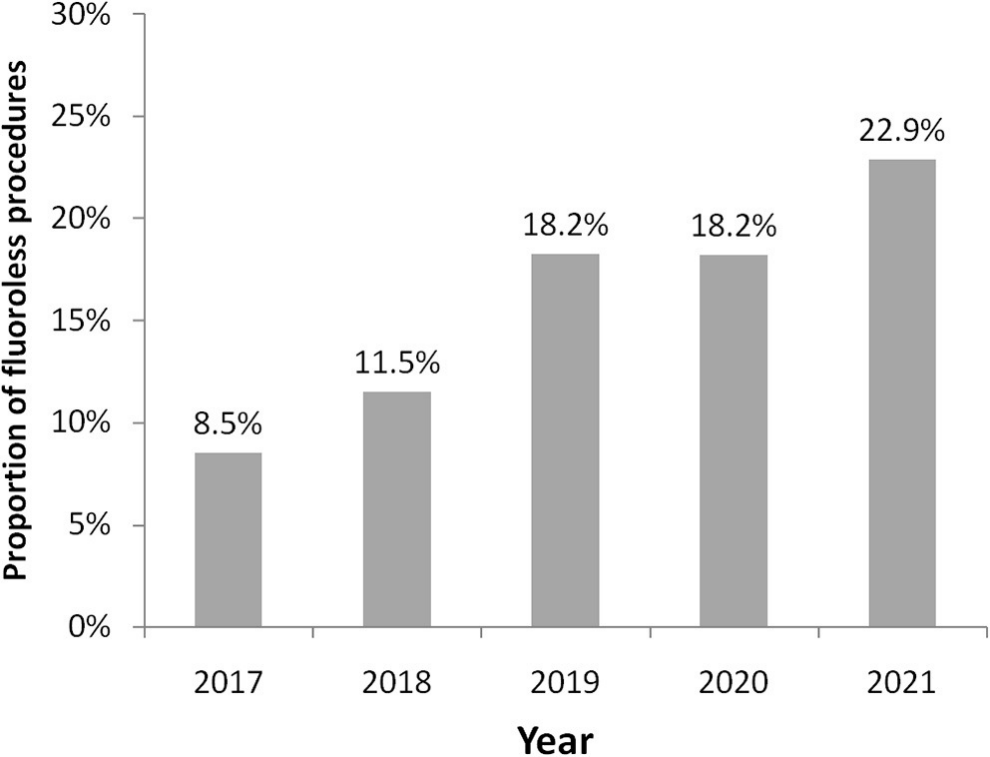
Proportions of fluoroless procedures by calendar year. Trend of fluoroless procedures.

During the study period, 1,853 procedures were performed (364 in the year 2017, 434 in 2018, 444 in 2019, 362 in 2020, and 249 during the first 6 months of 2021) for a total of 1,957 arrhythmias treated (some patients were treated for more arrhythmias during their procedure). Table 1 shows characteristics of patients treated with and without fluoroscopy. A fluoroless procedure, in comparison to the standard one, was more common in younger and female subjects. With the exception of diabetes mellitus and vascular disease, the prevalence of other comorbidities was lower in patients treated with a fluoroless intervention than those with fluoroscopy (Table 1). The proportion of fluoroless procedures involving a treatment of PVC or AP was greater than those performed with fluoroscopy, while AF and AFL were much more frequent with a standard approach. The relative frequency of AVNRT and VT within both groups was not different by study group (Table 1). The most frequently treated arrhythmia was AF (646; 33.0% of total arrhythmias) followed by AVNRT (644; 32.9%), AFL (215; 11.0%), VT (178; 9.1%), PVC (162; 8.3%), and AP (112; 5.7%). Right-sided AP, PVC, and VT were respectively 42.0, 58.0, and 23.6% of arrhythmias treated. Among procedures performed, 285 (15.4%) were fluoroless. Figure 2 displays the growth of procedures' proportion with a fluoroless approach over the study period (from 8.5 to 22.9% on yearly basis).

**Table 1:** Characteristics of patients treated with and without fluoroscopy.

	**Floroscopy**	**Fluoroless**	
	***N* = 1,568**	***N* = 285**	***p***
Age (years)	57 ± 14	42 ± 19	<0.001
Males	62.1%	46.0%	<0.001
Hypertension	40.4%	18.6%	<0.001
Diabetes mellitus	5.1%	4.2%	0.524
Chronic obstructive pulmonary disease	5.0%	1.4%	0.007
Severe renal dysfunction	3.3%	1.1%	0.042
Vascular disease	6.6%	4.9%	0.274
Previous myocardial infarction	6.4%	2.5%	0.009
History of heart failure	18.3%	13.0%	0.030
**Arrhythmias treated**			
AF	39.7%	8.1%	<0.001
AFL	12.5%	6.7%	0.005
AP	4.9%	12.3%	0.005
AVNRT	34.1%	38.2%	0.178
PVC	5.4%	27.4%	<0.001
VT	9.4%	10.9%	0.429

*Mean ± Standard Deviation or percentage*.

**Figure 3:**
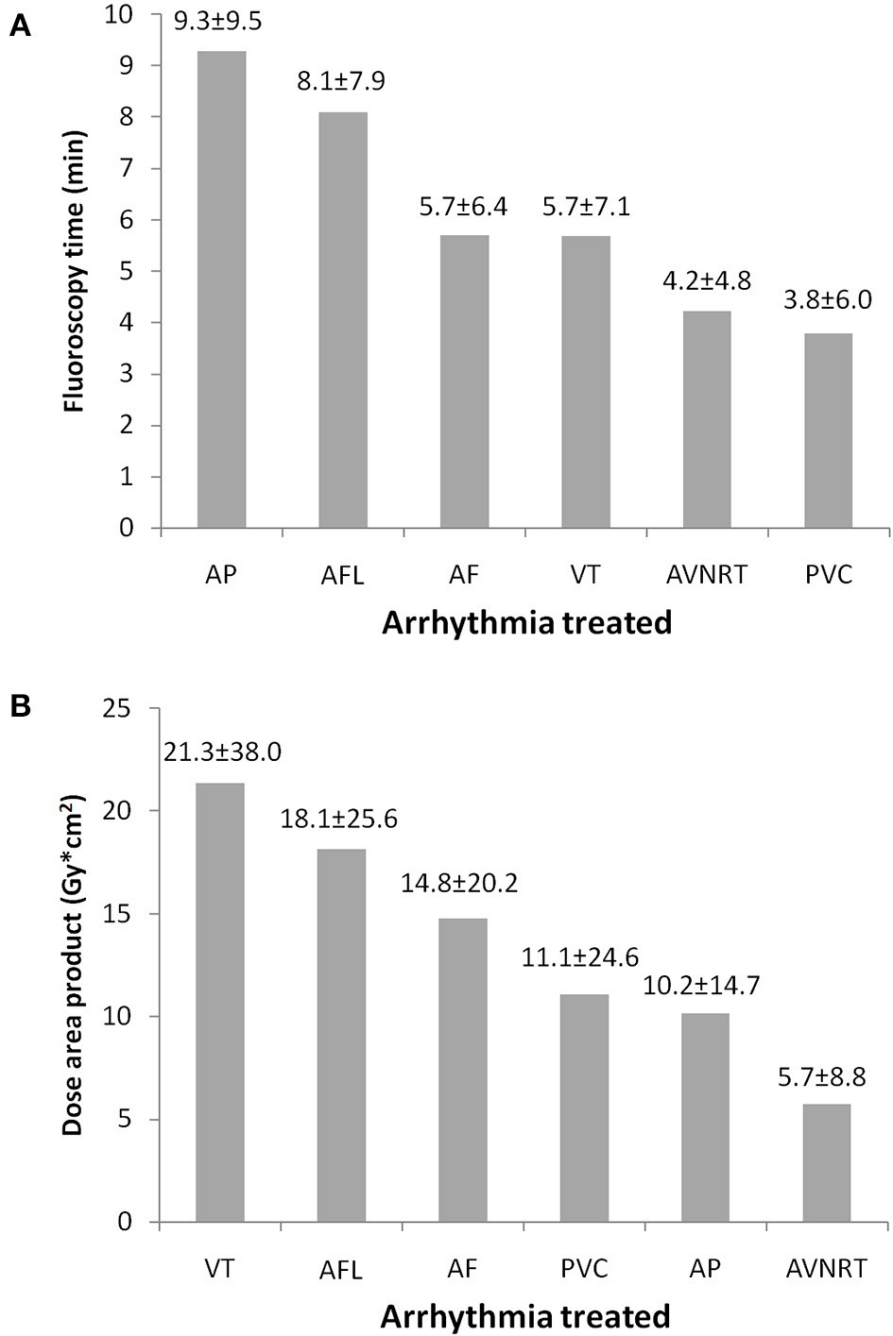
Fluoroscopy time **(A)** and dose area product **(B)** by arrhythmias treated in procedures with fluoroscopy use. Fluoroscopy time and dose area product. AF, atrial fibrillation; AFL, atrial flutter; AP, accessory pathways; AVNRT, atrioventricular nodal reentrant tachycardia; PVC, premature ventricular contraction; VT, ventricular tachycardia.

Table 2 shows characteristics of patients according to arrhythmias treated. Younger subjects more frequently underwent a fluoroless approach in all types of arrhythmias, with the exception of AF ablations for which this possibility was related to anatomic features (PFO). Women were more frequently treated with a fluoroless intervention for AP, PVC, and VT (Table 2). Procedure duration is found to substantially overlap between procedures with and without fluoroscopy, given that differences may be due to a greater or lesser presence of right or left forms of arrhythmias. A longer procedure duration was found in ANVRT fluoroless procedures and in PVC or VT fluoroscopy interventions (Table 2). Figure 3 shows fluoroscopy time and dose area product with regard to standard procedures (panel A and B). Fluoroscopy time was different among procedures: ~8 min in ALF, 6 in AF or VT, and 4 for AVNRT and PVC (Figure 3A). The mean of dose area product was higher in VT, AFL, and AF with lower values in PVC, AP, and AVNRT (Figure 3B).

**Table 2:** Patients' characteristics by arrhythmias treated.

	***N***	**Age (years)**	**Men/women**	**Procedure duration (min)**
**AF**				
Overall	646	59 ± 11	447 (69.2%)/199 (30.8%)	141 ± 50
Fluoroless	23 (3.6%)	57 ± 15	14 (60.9%)/9 (39.1%)	138 ± 30
Floroscopy	623 (96.4%)	59 ± 10	433 (69.5%)/190 (30.5%)	141 ± 51
		*p* = 0.360	*p* = 0.379	*p* = 0.808
**AFL**				
Overall	215	61 ± 12	159 (74.0%)/56 (26.0%)	125 ± 58
Fluoroless	19 (8.8%)	51 ± 11	14 (73.7%)/5 (26.3%)	133 ± 49
Floroscopy	196 (91.2%)	62 ± 12	145 (74.0%)/51 (26.0%)	124 ± 59
		*p* <0.001	*p* = 0.978	*p* = 0.496
**AP**				
Overall	112	31 ± 17	76 (67.9%)/36 (32.1%)	98 ± 43
Fluoroless	35 (31.3%)	20 ± 11	19 (54.3%)/16 (45.7%)	93 ± 39
Floroscopy	77 (68.8%)	36 ± 17	57 (74.0%)/20 (26.0%)	100 ± 45
		*p* <0.001	*p* = 0.038	*p* = 0.487
**AVNRT**				
Overall	644	50 ± 16	248 (38.5%)/396 (61.5%)	71 ± 34
Fluoroless	109 (16.9%)	38 ± 17	34 (31.2%)/75 (68.8%)	83 ± 37
Floroscopy	535 (83.1%)	53 ± 15	214 (40.0%)/321 (60.0%)	69 ± 33
		*p* <0.001	*p* = 0.085	*p* <0.001
**PVC**				
Overall	162	51 ± 16	93 (57.4%)/69 (42.6%)	130 ± 64
Fluoroless	78 (48.1%)	47 ± 15	34 (43.6%)/44 (56.4%)	106 ± 40
Floroscopy	84 (51.9%)	55 ± 16	59 (70.2%)/25 (29.8%)	152 ± 75
		*p* <0.001	*p* = 0.001	*p* <0.001
**VT**				
Overall	178	64 ± 16	156 (87.6%)/22 (12.4%)	180 ± 80
Fluoroless	31 (17.4%)	53 ± 19	22 (71.0%)/9 (29.0%)	122 ± 44
Floroscopy	147 (82.6%)	66 ± 14	134 (91.2%)/13 (8.8%)	193 ± 80
		*p* <0.001	*p* = 0.002	*p* <0.001

*Mean ± Standard Deviation and number (percentage) of patients*.

### Feasibility, Safety, and Short-Term Efficacy

All procedures were successful in both groups. Fluoroless feasibility was 100% without any conversion to standard procedure for non-AF-ablations. There were eight (0.4% of interventions performed) major complications: two femoral artery dissections (one in AVNRT; one in VT), three pericardial effusions without cardiac tamponade (one in AF; one in PVC; one in VT), and three with cardiac tamponade (one in ALF; one in PVC; one in VT). All complications were observed in the standard procedures with fluoroscopy, with the exception of the pericardial effusion without cardiac tamponade onset during a PVC fluoroless intervention. The rate of major complications between procedures with and without fluoroscopy was not different (0.45 vs. 0.35%; *p* = 0.999).

## Discussion

In our study we analyzed the experience of a high-volume electrophysiology center, where integration of X-Rays with EAM system has allowed a gradual reduction of the fluoroscopy exposure times. At the same time the growth of technical skill and the awareness of radiation damage have led to a progressive increase of ablative procedures without using fluoroscopy at all. This latter approach in our experience has proved effective, safe, and feasible to ablate almost all types of arrhythmias, as already demonstrated in previous papers (11, 12). This is a large study involving a very heterogeneous set of recent procedures performed with the conventional fluoroscopic ablations or by using the zero fluoroscopic approach. The novelty of our register compared to others is exactly the relevant number of interventions and the heterogeneity of the cases. We have shown that a single center strongly oriented to reduce fluoroscopy to zero can perform ablation procedures with fluoroless method in definitely all the possible arrhythmias to be treated.

The idea of performing fluoroless ablations initially took hold in the field of interventional electrophysiology of the pediatric age: performing ablation of reentry supraventricular tachyarrhythmias without X-Ray in children appeared immediately as an interesting opportunity (13). Simultaneously, several studies began to show that, even in adults, a non-fluoroscopic approach was possible and safe for catheter ablation of supraventricular arrhythmias (14, 15). There has also been described isolated experiences of fluoroless ablation for ventricular and more complex arrhythmias (16–18). In some cases of complex procedures, however, it is currently not technically possible to do fluoroless ablations. In fact, in patients with cardiac implantable electronic devices, it is necessary to use fluoroscopy to visualize the catheters present in the heart chambers, while the operator moves ablation catheters. We lastly need X-Rays also in complex procedures like electrical storm ablations with cardiopulmonary support (19). In recent years, data from some hospitals have been published to highlight their volume of activity and their skill in performing ablations without fluoroscopy in various arrhythmias (20–22). In our experience we point out that the ablative procedure with the integration of X-Rays and EAM leads to a reduction in the average time of fluoroscopy compared to the past, as already known (23). The use of contact force-sensing catheters has improved the information that EAM provides during ablation, further reducing the need for fluoroscopy (24). Intracardiac echocardiography enhances direct visualization of catheter against the tissue, giving a better sense of contact. In our electrophysiology laboratory we have used contact-force sensor catheters since 2017, so we have been able to limit the use of intracardiac echocardiography in our routine. This ultrasound technique, which has a significant economic cost, is reserved for ablations of ventricular ectopic beats from papillary muscles or other challenging procedures. Transesophageal echocardiography is also a useful tool, particularly for safe transeptal puncture, but it requires general anesthesia. We performed procedures in conscious sedation, so unfortunately, we could not use it at all.

We noted that, to go fluoroless, it is important to have anatomical reference points on the electroanatomic map to work by continuously integrating electrophysiological potentials with the anatomical and structure information that EAM provides.

The advent of new technologies has led us to progressively increase fluoroless procedures over the years, so for most of the arrhythmias, we observed a trend of a progressive increase in the percentage of procedures without scope, that for AVNRT reaches almost 40% of the total ablations. For AF we have not recorded an increase in procedures, since in this arrhythmia to perform fluoroless ablation depends on the possibility of being able to pass through the PFO into the left atrium. However, the use of the technique we previously described for transseptal puncture has allowed us over the years to make this procedure a “near-zero fluoroscopy ablation” (10). In 2020, the volume of activity of our center has been slightly reduced due to the coronavirus pandemic and, in particular, urgent and non-deferrable ablative procedures were favored. For this reason, there was no substantial increase in the percentage of procedures without fluoroscopy, which remained stable compared to the year 2019. In the first 6 months of 2021, with the gradual resumption of the normal rhythm of work, the growth trend of procedures without X-Rays clearly regained.

However, in our data overall from 2017 to today, the percentage of fluoroless procedures is 15% of all ablations carried out; that is considerably higher than the percentage that emerged from the European multicenter registry of interventional electrophysiology (7). In particular, the progressive and important increase of this percentage over the years from 9 to 23% means that a center with a high volume of ablations, strongly oriented toward zero X-Rays, can carry out a quarter of its procedures with this approach.

There are ablations of some arrhythmias (such as AVNRT or AFL or AP) for which the use of the EAM system is a plus, although the costs of the procedure. There are several operators who have alternated over the study years in our electrophysiology laboratory. Among these operators, four were mid-career with more than 5 years of experience in electrophysiology and a volume of 20–40 procedures per month, while one is a mentor with more than 20 years of experience in electrophysiology and more than 40 procedures per month.

In our laboratory, regardless of which operator carrying out the procedure, the criteria used from time to time to decide on a fluoroless ablation were essentially the evaluation of the age and sex of the patients; that is in line with evidence already demonstrated (25). In general, we prefer to avoid the use of X-Rays in younger people in general and in women, particularly if of childbearing age. It has already been proven that the risks of cancer incidence and mortality decreased with aging and is always higher for female patients at the same age of intervention (26).

Ionizing radiations are carcinogenic and teratogenic both for patients and for medical staff. Biological effects of X-Rays are classified as stochastic (carcinogenic and genetic effect) and deterministic. Stochastic effects have a “linear non-threshold” model: any small amount of radiation leads to an increase in cancer risk without any threshold, and the probability increases linearly with increasing radiation dose. For deterministic effects (e.g., skin injuries, cataracts, etc.), there is a “threshold of dose:” below this value, the effect is not produced, and the severity increases with the dose (27). We calculated on the basis of the mean fluoroscopy times observed in our procedures with X-rays that the total number of fluoroless ablations since 2017 has allowed to save patients and the healthcare team from exposure of an average of 5 h of fluoroscopy for year; that means avoidance dose area product of 650 Gy^*^m^2^ per year. This potentially achieves a total saving of 25 h of scope and 2,955 Gy^*^m^2^ of dose area product over the years of interest. There are moreover physical damages for the operators, mainly of an orthopedic and neurosurgical nature, which derive from the prolonged use of protective lead apparel. Obviously, procedures without fluoroscopy do not require the use of these devices, preventing their orthopedic and ergonomic consequences (28).

All procedures that started fluoroless have been successful. Only in AF ablations were the usage of X-Rays dependent on the possibility of going through PFO. During this procedure we do not use routinely intracardiac echocardiography, therefore we cannot always avoid the use of X-Rays if there is PFO. The rate of major complications was very low (0.4%), compared to the complication rate described in literature (29, 30), without differences between ablation with and without fluoroscopy; this means that the latter approach does not increase procedural risks. We observe, furthermore, substantial equality in duration of procedure between fluoroless or traditional ablations, like in other experiences (22, 31).

The acute success of zero X-Rays arrhythmia ablation that we reported requires a long-term outcome assessment in terms of recurrences of arrhythmia and complications. In a recent study, 266 patients who underwent ablation without the use of fluoroscopy were followed-up over a period of 6 years. The acute success was 100% and the chronic success was 90.8% with a new post-ablation arrhythmia that occurred in 7.7% of the sample (32). In another study comparing long-term outcomes of near-zero radiation ablation of paroxysmal supraventricular tachycardia with fluoroscopy-guided approach, cardiac ablation guided by EAM systems provided better results compared with conventional fluoroscopic ablation (33). According to our data, based on a large number of various arrhythmias treated, the complete elimination of fluoroscopy during catheter ablation does not reduce patient safety with an acute success comparable to conventional procedures. Further studies are needed in order to assess long-term outcomes and compare them among approaches.

Today in an electrophysiology laboratory, operators face a choice in procedures that could require an EAM: save the patient from X-Rays at the expense of an increase in the cost of the procedure. It has been proven very well that spending money today can mean saving money tomorrow in terms of gain in health and saving care costs (26). Conversely there are other procedures in which EAM is used in any case, such as PVC, VT, or AF ablation. In the latter cases, the goal is to take full advantage of the mapping system that is already being used and to go fluoroless. In these procedures using or not using X-Rays is a matter of habit and training. It is, however, recommended to start with right-sided ablations and then proceed with more complex procedures. Specific training programs are needed to instruct new operators on the development of these skills (34, 35).

## Study Limitations

This was a retrospective single center register subjected to inherent limitations. The study was not a randomized clinical trial and reported data without a matching procedure to pair patients. The decision for a fluoroless ablation was made subjectively by operators. We did not make a formal evaluation of the learning curve of the various operators of the center, because some of them had already used zero X-Rays approach before 2017. For AF procedures, feasibility depends on PFO presence and difficulties to go through without an intra-procedural echocardiography guide. The large sample size was a point of strength for conclusions regarding safety and short-term efficacy. The absence of mid- or long-term clinical follow-up was the main limitation of our research. Specifically designed comparative studies could better investigate differences between procedural techniques.

## Conclusion

An electrophysiology center with a high volume of procedures at the present time should be strongly oriented to a fluoroless approach, limiting the use of X-Rays only to ablations in which it is still really indispensable. We firmly believe that the continuous improvement of available technologies will give in the future the possibility to invert the current proportion between procedures with and without fluoroscopy, making those with X-rays a minority of all electrophysiological activity.

## Data Availability Statement

The original contributions presented in the study are included in the article/supplementary materials, further inquiries can be directed to the corresponding author/s.

## Ethics Statement

The studies involving human participants were reviewed and approved by Azienda Universitaria Ospedaliera Consorziale - Policlinico, Bari, Italy. The patients/participants provided their written informed consent to participate in this study.

## Author Contributions

FT and MG contributed to conception and design of the study. FT and PG performed the statistical analysis. FT wrote the first draft of the manuscript. All authors contributed to manuscript revision, read, and approved the submitted version.

## Conflict of Interest

MG has an agreement with Biosense Webster regarding the development of new technologies not related to this article. The remaining authors declare that the research was conducted in the absence of any commercial or financial relationships that could be construed as a potential conflict of interest.

## Publisher's Note

All claims expressed in this article are solely those of the authors and do not necessarily represent those of their affiliated organizations, or those of the publisher, the editors and the reviewers. Any product that may be evaluated in this article, or claim that may be made by its manufacturer, is not guaranteed or endorsed by the publisher.

## Abbreviations

3D: three-dimensional
AF: atrial fibrillation
AFL: atrial flutter
AP: accessory pathways
AVNRT: atrioventricular nodal reentrant tachycardia
EAM: electroanatomical mapping
PFO: patent foramen ovale
PVC: premature ventricular contraction
VT: ventricular tachycardia.
